# Digital Advance Care Planning for Dialysis Patients - Usability and Acceptability

**DOI:** 10.1016/j.ekir.2025.08.038

**Published:** 2025-09-03

**Authors:** Chetna Malhotra, Chandrika Ramakrishnan, Joshua Lakin, Jason Chon Jun Choo, Alethea Yee

**Affiliations:** 1 Lien Centre for Palliative Care, Duke-NUS Medical School, Singapore, Singapore; 2 Program in Health Services and Systems Research, Duke-NUS Medical School, Singapore, Singapore; 3 Department of Psychosocial Oncology and Palliative Care, Dana Faber Cancer Institute, Boston, Massachusetts, USA; 4 Department of Renal Medicine, Singapore General Hospital, Singapore, Singapore; 5 National Kidney Foundation Singapore, Singapore, Singapore; 6 Division of Supportive and Palliative Care, National Cancer Centre Singapore, Singapore, Singapore

**Keywords:** acceptability, advance care planning, dialysis withdrawal, digital interventions, end-stage kidney disease

## Introduction

End-stage kidney disease (ESKD) progression often presents patients, their informal care partners, and health care providers (HCPs) with complex care decisions particularly regarding dialysis continuation. Advance care planning (ACP) conversations are often not initiated, leaving many patients with ESKD without sufficient information about the uncertain trajectory of their illness from their HCPs. As a result, they may not perceive themselves as having a terminal illness, may maintain optimistic expectations about the benefits of dialysis, and may view dialysis discontinuation as a form of medical abandonment.^1^ HCPs, in turn, often have limited understanding of what matters most to patients when making decisions about continuing or discontinuing dialysis.^2^ Ongoing ACP conversations that educate patients and their care partners about the illness, clarify their values and goals of care, and prepare all parties for future dialysis-related decisions are essential.^3^, ^4^, ^5^ To improve ACP engagement, we developed *My Voice*, a novel digital intervention, tailored for patients with ESKD on dialysis and their informal care partners. Although discontinuation of dialysis is an important focus, *My Voice* is intended to support ACP more broadly across the illness trajectory. This study’s objective is to assess the tool’s usability and acceptability among patients on dialysis, their informal care partners, and HCPs. This testing is critical for ensuring the tool’s relevance, ease of use, and the likelihood of adoption in routine care.

*My Voice* is a theory-driven web-based intervention guided by the Capability Opportunity Motivation Behaviour model for behavior change and the Educate-Share-Prepare framework for ACP.^3^^,^^6^ Adapted from a similar platform developed for patients with heart failure, also called *My Voice*,^7^ the initial prototype was developed based on user-centered design principles and included patient and care partner modules in 2 languages, English and Mandarin (Supplementary Figure S1). The patient module integrates educational videos (on kidney failure, dialysis, and what happens when dialysis is stopped), a value clarification exercise (to identify the patient’s values and goals of care), option to name surrogate(s), a sharable *My Voice* summary document listing their chosen values and goals of care (Supplementary Figure S2), and phone text reminders to revisit *My Voice* to update their goals of care. The care partner module includes educational videos (on kidney failure, dialysis, and what happens when dialysis is stopped), coaching videos (explaining surrogate’s role and supporting their loved one), and access to patient’s *My Voice* summary document if named as a surrogate. We describe the development of *My Voice* in the Supplementary Methods.

Testing included a mixed-methods design with moderated think-aloud sessionsS1^,^S2 that were audio-recorded, transcribed, and thematically analyzed,S3 and surveys to assess system usability and acceptability.^8^^,^^9^ Qualitative feedback helped understand problems related to the tool’s usability and acceptability, and informed iterative refinements to *My Voice*. A full description of the methods is found in the Supplementary Methods. The study received approval from the Institutional Review Board of the National University of Singapore, (Ref no NUS-IRB-2021–749) and was conducted in accordance with the relevant guidelines and regulations. Written informed consent was obtained from all participants.

## Results

Twenty-four participants including 9 patients on dialysis, 5 informal care partners, and 10 HCPs participated. Participants had a mean age of 57 years (SD: 14.9; range: 29–78), and 54% were females. In Supplementary Table S1, we describe participant characteristics in detail. Usability and acceptability were assessed using the system usability scale, and questions adapted from the Acceptability Rating Scale for Decision-Aids.^8^^,^^9^ The mean system usability scale score was 76.2 (SD: 15.3; range: 45–97.5), with all participant groups achieving a mean score greater than the standard cut off of 68, indicating good usability (Supplementary Table S2).

Most participants (80%) rated the website as good or excellent. High acceptability was reflected in responses to several key questions—92% felt the amount of information was “just right,” 91% considered it useful for making future decisions about dialysis, and 96% would recommend it to others (Supplementary Table S3).

During qualitative interviews, participants described *My Voice* as user-friendly and informative with clear accessible language and interactive elements that enhanced learning. The themes indicated that *My Voice* provided supportive information and reassurance, encouraged patient-centered, empathic communication regarding values and goals of care, and facilitated discussions on dialysis continuation or discontinuation. Expert-led videos provided credible education, particularly on dialysis decisions, reassuring patients about continued care. In Table 1, we present the themes with illustrative quotes. Suggestions for improvement included enhancing aesthetics and navigation, offering the website in other local languages, and delivering *My Voice* as a guided tool to enable better implementation and adoption.

**Table 1:** Themes from user-feedback with illustrative quotes

Theme	Illustrative quotes
Supportive information and reassurance	“Very useful. It is something that gives extra knowledge. Even if you go to the doctor, the doctor is unable to explain so much but having this thing (website) and videos, at least you can learn something.” PT04 “I feel like this website is helpful to patients and family, give the caregiver a lot of help, to understand. Otherwise, they will not understand. …It also gives me more knowledge, so I’m happy I can share what I know with others.” CG05 “I mean in terms of they want to stop dialysis, what they need to do and if you stop dialysis, there is reassurance in the video (no 2). They will try to manage your symptom and all this. And then they also teach people when you are becoming worse, you cannot speak yourself, what you need to do. So, the reassurance continues that care is there and what they need to do…..I think it will be a very useful platform to have on both clinical and the community setting.” HCP07
Patient-centric tool encouraging ongoing reflection and communication of values and goals of care	“Not many people may realize it until answering the questions, “What I want?” …After answering these, you start asking yourself, “Is this what I want?” (then) you somehow have a clearer picture. And it [My Voice document] helps someone to basically understand their issue … If you see the doctor, it is just like one kind of talk not very valid, consolidated information.” PT05 “Because normally when that thing happens, people sometimes get confused what to do. So once the patient has already informed their close relatives, brother-sister, then easy for them to execute whatever needed when the worst comes.” CG02 “So, it's actually quite crucial because it touches on topics that you will not discuss about in your regular life. Like, you know, talk during dining tables, going for travel instead. You wouldn’t have this conversation at all. And when you have this kind of conversations, actually it is at stage, which is too late. I think it is quite important.” HCP04
User-friendly, interactive needing improved aesthetics and navigation	“This one (sentence) I can read. Small one (font) cannot read, need spectacle.” PT03 “The page (My Voice document) is okay but this one I think you need to make it darker a bit.” CG04 “I think it's very good because for people like me, I like to see videos like to do quizzes. I think it's more interactive compared to just like sitting down and just talking to a person for half an hour to an hour.” HCP08
Perceived implementation challenges	“All the videos are clear. If possible, can play to the all the community….Maybe put up mosques, churches, Chinese temple or community center. So, that more people can see (videos).” PT08 “Some kind of translation. Like the Malays like us are not very fluent and don’t understand the language. Language is a barrier especially for people my age. So, I think it needs to be translated.” CG04 “Do together, do assisted (by HCP). They (patients) must have a bit of education and IT smart able to use this gadget.” HCP06

CG, care giver; HCP, health care provider; PT, patient.

**Figure 1:**
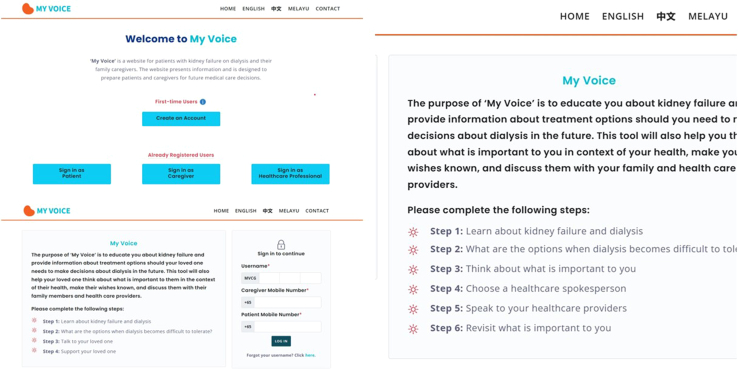
‘My Voice’ website sample pages.

We iteratively revised *My Voice* based on participant feedback. Major changes included addition of an HCP-assisted module, an updated video on dialysis discontinuation incorporating information on palliative dialysis, inclusion of a Malay language option, enhancements to navigation and aesthetics, and refinements to the value clarification questions and response options (Supplementary Table S4). In Figure 1 we illustrate pages from *My Voice*.

## Discussion

*My Voice* has many strengths. It is one of the first disease-specific ACP tools tailored to the unique needs of individuals with ESKD on dialysis and their informal care partners. The tool integrates education on dialysis withdrawal and palliative dialysis, includes a value clarification exercise to support patient choice, and emphasizes a key component of ACP—identification of a surrogate decision maker—making it distinct. *My Voice* shows high usability and acceptability; and incorporates patient, informal care partner, and HCP feedback in its development. Although dialysis discontinuation discussions are challenging, *My Voice* demonstrated high acceptability because of its balanced and supportive approach that reassures patients while prompting ongoing value-based reflections.

*My Voice* recognizes Singapore’s collectivistic decision-making culture and the integral role that informal care partners play in medical decision-making by incorporating a care partner module with videos guiding surrogate decision-making. With patient consent, appointed surrogates have access to the patient’s *My Voice* summary document, a simplified advance care plan incorporating patient’s values as well as current and future care goals, including dialysis. In parallel, *My Voice* empowers patients to reflect on their values and care goals, enabling control of their decision-making. This dual approach—targeting both informal care partners and empowering patients— bridges the communication gap between them. This is crucial, because our previous qualitative study revealed that patients are often excluded from decisions about their own care, with informal care partners dominating the process.^1^

*My Voice* ensures inclusivity for patients with low digital literacy by incorporating an HCP-assisted patient module that allows HCP-facilitated navigation and completion, potentially reducing the time required for ACP conversations and documentation. In addition, *My Voice* accounts for the possibility that patients may change their minds over time, offering options to revisit and systematically triggering phone reminders to update *My Voice* document. This feature, which allows for ongoing reflection and adjustments, is unique and sets *My Voice* apart from other tools.

This study adds to the evidence base of interventions targeting ACP focusing on dialysis decision-making at the end of life. The strength lies in the use of a mixed-methods, iterative design and user-centered principles, which were essential for enhancing user satisfaction and acceptability. Involvement of relevant end users, namely patients, caregivers, and HCP contributed to important outcomes of the intervention development. However, there are some limitations. Although the sample size was adequate to achieve thematic saturation, it was too small to discern variations across age, gender (male/female), and other sociodemographic characteristics. Further, the study patients were from community dialysis centers and may not represent the general ESKD population.

### Conclusion

*My Voice*, tailored to the unique needs of individuals with ESKD on dialysis and their caregivers, shows good usability and high acceptability. It addresses a critical gap in ACP and fosters informed discussions about future health care decisions about dialysis discontinuation. *My Voice* holds promise for wider implementation to improve ACP engagement in dialysis care and a randomized controlled trial is underway to test its impact on ACP engagement.

## Disclosure

All the authors declared no competing interests.

### Funding

The study was supported by the Venerable Yen Pei-NKF Research Grant from National Kidney Foundation (NKF) Singapore (grant no NKFRC/2021/01/01). The funding agency had no role in the design of the study or the interpretation of the findings.

## Data Availability Statement

Data from the study is available on reasonable request from the corresponding author subject to approval from the institution, funder, and ethics review board.

## Supplementary Material

Supplementary File (PDF) **Supplementary Methods. Supplementary References. Figure S1.***My Voice* website modules and flow. **Figure S2.** Sample *My Voice* summary document. **Table S1.** Participant characteristics. **Table S2.** System usability scores by participant group. **Table S3.** Acceptability ratings of *My Voice*. **Table S4.** Changes made to *My Voice*.
