# Supplementary material for: Digital Advance Care Planning for Dialysis Patients - Usability and Acceptability
Source: Kidney Int Rep. 2025 Sep 3;10(11):4073–6. doi: 10.1016/j.ekir.2025.08.038 (PMC12640010; doi:10.1016/j.ekir.2025.08.038)
Supplement: Supplementary File (PDF) — Supplementary Methods. Supplementary References. Figure S1.My Voice website modules and flow. Figure S2. Sample My Voice summary document. Table S1. Participant characteristics. Table S2. System usability scores by participant group. Table S3. Acceptability ratings of My Voice. Table S4. Changes made to My Voice. [file mmc1.pdf]

SUPPLEMENTARY MATERIALS

Contents

Supplementary Methods 2

A. *My Voice* website development 2

B. *My Voice* patient module description 3

C. Usability and acceptability testing 7

Supplementary References 8

Supplementary Tables 8

Supplementary Table 1: Participant characteristics 8

Supplementary Table 2: System Usability Scale score overall and by participant group 9

Supplementary Table 3: Acceptability ratings of *My Voice* 10

Supplementary Table 4: Changes made to 'My Voice' as suggested by participants 11

Supplementaty Figures

Supplemetary Figure1...2

Supplemetary Figure 2...7

Supplementary Methods

A. *My Voice* website development

The initial prototype includes patient and caregiver modules in two languages - English and Mandarin. The website adapted from a similar platform developed for patients with heart failure, also called *My Voice* (ref 7), included one additional step to educate patients and care partners about what happens when dialysis is stopped.

My Voice consists of a series of videos, value-clarification exercise (VCE) and knowledge quiz. The video scripts for both patient and caregiver modules were drafted by the study team in English and then translated into Mandarin. A professional company developed the videos, with healthcare providers from the study team—nephrologists and social workers—serving as narrators. We then dubbed the videos into Mandarin and added subtitles.

The patient module has 6 ordered steps – (1) understanding kidney failure and dialysis, (2) learning what happens when dialysis is stopped, (3) reflecting on values and goals of care, (4) choosing a surrogate, (5) speaking to health care providers (HCPs), and (6) revisiting *My Voice*. A summary *My Voice document* generated at the end is shared with patients, designated surrogates and HCPs. Automated phone reminders remind patients to revisit *My Voice* to update their values and goals. Informal care partners who are nominated as surrogate(s) receive phone notification to view *My Voice* and their loved one's *My Voice* document. The informal care partner module has 4 steps – (1) understanding kidney failure and dialysis, (2) learning what happens when dialysis is stopped, (3) talking to loved one and (4) supporting loved one.

VCE questions followed Step 3 video in the patient module were developed as multiple choice format based on language from our previous study, (ref 7) adding two questions specific to dialysis- one on how dialysis is currently helping patients and another on their dialysis decisions if their health worsens in the future.

Knowledge quiz followed the educational videos in Steps 1 and 2 in both patient and care partner modules. It included questions about kidney failure, dialysis, options when dialysis becomes difficult to tolerate and supportive care. **Supplementary Figure 1** gives a snapshot of the website modules and its flow.

Supplementary Figure 1: My Voice website modules and flow

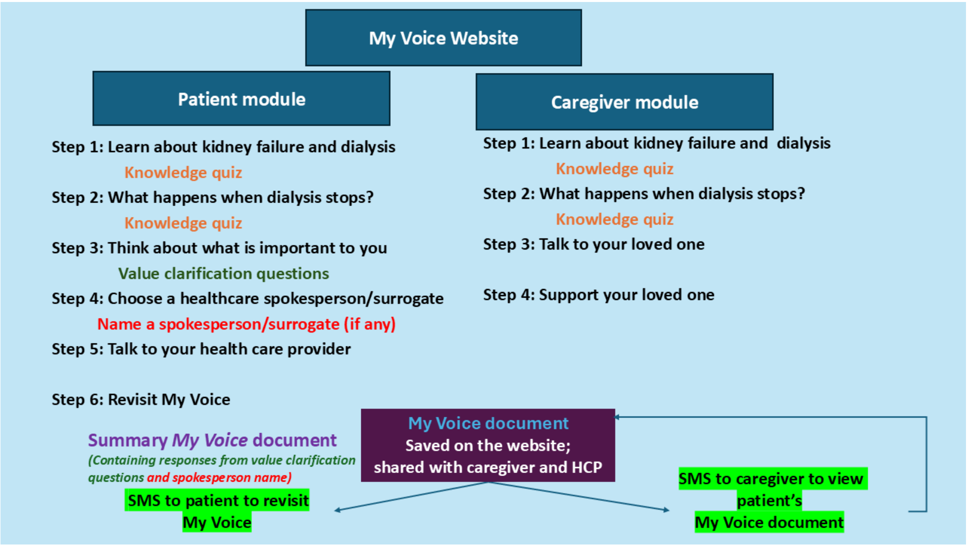

B. *My Voice* patient module description

The website is under production, hence we are unable to share the website link.
Here, we present information in the patient module consisting of 6 steps with links to the respective videos, knowledge quiz, value clarification exercise and a sample *My Voice* summary document.

Step 1: Learn about kidney failure and dialysis
https://www.youtube.com/watch?v=GeRRrKuqTEo&list=PLqYLXdZ4Z71G35FgBvV4rtpim179WsGul&index=1

Knowledge quiz

Q1. Kidney failure will get better over time.

- True
- False
- Unsure

Q2. Dialysis will cure my kidney condition completely.

- True
- False
- Unsure

Q3. Dialysis is a treatment that can never be stopped**.**

- True
- False
- Unsure

Step 2: What are the options when dialysis becomes difficult to tolerate?
https://youtu.be/Ky2L7eRxbQg?si=YlvqFxq9ROani7V5

Knowledge quiz
Q4. Palliative or comfort dialysis is when the frequency of dialysis per week is reduced.

- True
- False
- Unsure

Q5. Once a person stops dialysis treatment, the person's symptoms can be managed to keep him/her comfortable

- True
- False
- Unsure

Step 3: Think about what is important to you
https://www.youtube.com/watch?v=E0dXQS3411M&list=PLqYLXdZ4Z71G35FgBvV4rtpim179WsGul&index=4

Value clarification exercise

Q1. What is most important to you right now?

(Select up to 3, minimum 1)

- Being able to spend time with my family and friends
- Being independent in my daily life (eg. walking, bathing etc)
- Being able to follow my faith or spiritual beliefs
- Being able to do things I enjoy (eg: hobbies, traveling, working)
- Having less symptoms (eg less pain, breathlessness etc)
- Making a legacy (something that is passed on, monetary or non-monetary)
- Anything else, specify______
- Anything else, specify______
- Anything else, specify______

Q2. Currently, dialysis is helping you:

(Select up to 2, minimum 1)

- Reduce symptoms (eg breathlessness, swelling, etc)
- Fulfill duties towards my family
- To continue working
- Live longer
- Be supported till I get a kidney transplant
- Anything else, specify______
- Anything else, specify______

Q3. When you think about your health getting worse, what worries you most?

(Select up to 5, minimum 1)

- Having symptoms (e.g., breathlessness, swelling, pain, tiredness etc.)
- Becoming dependent for my basic activities of daily living (e.g., eating, bathing, dressing, toileting etc.)
- Being a burden on my family
- Not having someone to take care of me
- Being unable to spend time with my family and friends
- Paying for treatments and daily expenses
- Having a sense of uncertainty about the future
- Being hospitalized
- Receiving more treatment
- Receiving less treatment
- Dying
- Anything else, specify_________
- Anything else, specify_________

Q4 (a). If your health condition gets worse, what matters most to you?

(Please choose only 3)

- o Being symptom-free and comfortable as much as possible
- o Being able to think and make decisions myself
- o Being able to communicate (eg. Speak)
- o Being able to fulfil my family duties
- o Having family support
- o Doing my daily activities independently (eg bathing, eating, walking)
- o Getting all possible treatments to live longer, at any cost
- o Not spending too much money on my treatments
- o Anything else, specify_________
- o Anything else, specify_________

Q 4 (b). You have selected these goals as your top 3 goals. Now rank them in order of importance, with the most important goal being ranked first.

(Show patients their top three goals that they selected)

______Goal 1_________

- o 1st
- o 2nd
- o 3rd

_______Goal 2________

- o 1st
- o 2nd
- o 3rd

___Goal 3____________

- o 1st
- o 2nd
- o 3rd

Q5. In the future, you may consider reducing or stopping dialysis if you are:

(Select up to 3, minimum 1)

- o Completely bed-bound
- o Unable to think and take own decisions
- o Complete with family responsibilities and life goals
- o Alone with no one to take care
- o Diagnosed with another serious illness
- o Unable to communicate with others
- o No, will continue dialysis no matter what
- o Anything else, specify_________
- o Anything else, specify_________

Step 4: Choose a healthcare spokesperson
https://www.youtube.com/watch?v=A05_wMbgLtY&list=PLqYLXdZ4Z71G35FgBvV4rtpim179WsGul&index=5

https://www.youtube.com/watch?v=bf3X6d2DHy0&list=PLqYLXdZ4Z71G35FgBvV4rtpim179WsGul&index=6

Step 5: Speak to your healthcare providers
https://www.youtube.com/watch?v=JuN78c_CNWA&list=PLqYLXdZ4Z71G35FgBvV4rtpim179WsGul&index=7

Step 6: Revisit '*My Voice*'
https://www.youtube.com/watch?v=OoTSzN8Kgy4&list=PLqYLXdZ4Z71G35FgBvV4rtpim179WsGul&index=7

At the end of Step 6 patients can edit and submit their *My Voice* summary document that captures the responses from the VCE questions (Step 3) and name(s) of healthcare spokesperson/ proxy if declared (Step 4). A sample summary *My Voice* document is shown in **Supplementary Figure 2**.

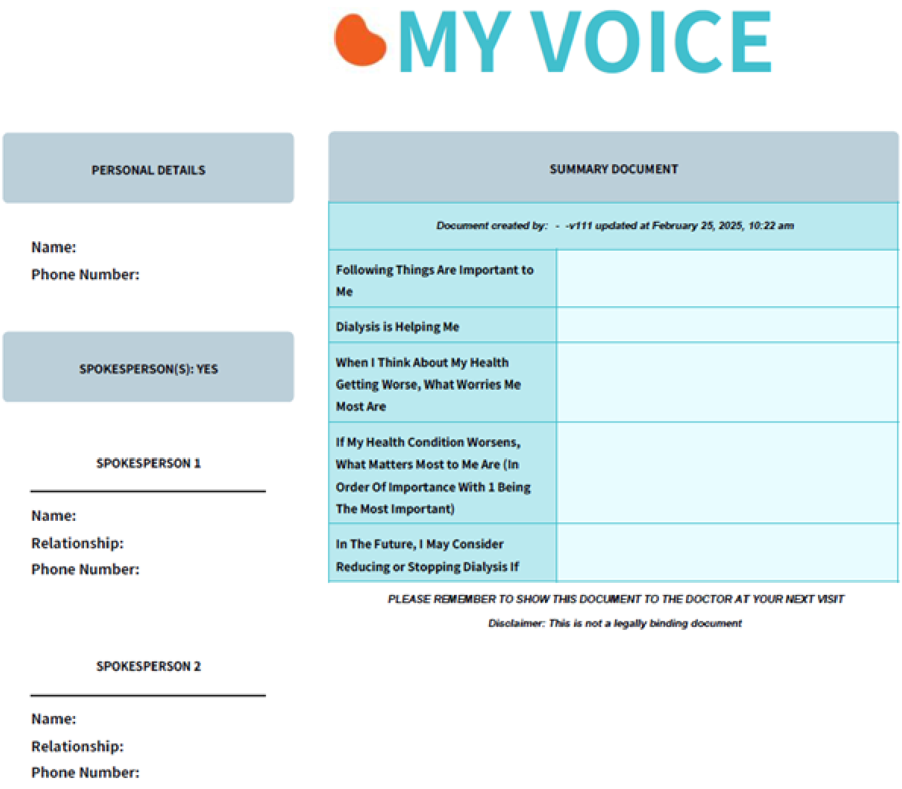

MY VOICE

PERSONAL DETAILS

Name:
Phone Number:

SPOKESPERSON(S): YES

SPOKESPERSON 1

Name:
Relationship:
Phone Number:

SPOKESPERSON 2

Name:
Relationship:
Phone Number:

SUMMARY DOCUMENT	
Document created by: - -v111 updated at February 25, 2025, 10:22 am	
Following Things Are Important to Me	
Dialysis is Helping Me	
When I Think About My Health Getting Worse, What Worries Me Most Are	
If My Health Condition Worsens, What Matters Most to Me Are (In Order Of Importance With 1 Being The Most Important)	
In The Future, I May Consider Reducing or Stopping Dialysis If	

PLEASE REMEMBER TO SHOW THIS DOCUMENT TO THE DOCTOR AT YOUR NEXT VISIT

Disclaimer: This is not a legally binding document

Supplementary Figure 2: Sample *My Voice* summary document

C. Usability and acceptability testing

We used a descriptive qualitative approach to gather feedback and enable iterative development of *My Voice* through semi structured in-depth interviews.

Setting and participants

We recruited a purposive sample of patients undergoing dialysis from the National Kidney Foundation Singapore (NKFS) dialysis centres, their caregivers, and HCPs. Eligibility criteria for patients include- being 21 years or older, currently undergoing dialysis, cognitively intact (assessed by an abbreviated mental test or by a physician or from medical records), agreeable for audio recording, and to satisfy one of the following- a) estimated to have a prognosis of less than 12 months (as determined by a treating physician using the surprise question- *"Would I be surprised if this patient died in the next 12 months?)* or b) tolerating dialysis poorly (physically or mentally) as identified by a treating physician, or c) patients who voiced to discontinue dialysis.

Caregivers of eligible patients were approached for participation with the following eligibility criteria - a) adults 21 years or older, b) provided informal care or ensured provision of care, and/or main decision-maker for the patients with no expectation of financial compensation, c) able to understand any of the English or Chinese and d) willing to use a web-based intervention. We recruited caregivers even if the corresponding patient did not participate in the study. Eligible patients and their caregivers were contacted in person or over telephone to schedule the interviews. The interviews were conducted in the health care facility or at a convenient place (participant's homes).

Health care providers involved in the care of dialysis patients (for at least 6 months) – nephrologists, nurses, renal counsellors and medical social workers practicing in NKFS and department of renal medicine from a large public hospital were invited to participate via email. On confirmation, they were interviewed in person at the health care facility.

Among 14 patients, one refused and 4 were medically unfit for interviews. Hence, we interviewed a total of 9 patients. Among 6 caregivers approached, one declined participation. All 10 HCPs approached, agreed for interviews.

Prior to interviews, all participants provided written informed consent, and the study received ethics approval from the National University of Singapore, Institutional Review Board.

Procedure

We conducted a moderated usability testing (S1), during which we asked each participant to browse the *My Voice* website either independently or with guidance from an experienced qualitative researcher (co-first author CR) or a study team member trained in interview methods. Using a topic guide developed by the authors, we followed concurrent 'think-aloud' method for each step asking open-ended questions to participants to vocalise their experiences using the website and gathered suggestions for its improvement. (S2) Participants were briefed on the purpose of the interviews prior to the start of the sessions. There were no other people present besides the interviewer and the participant, and the participants had no dependent relationship with the interviewers. We audio-recorded and transcribed the interviews for in-depth analysis and took field notes on areas needing revision. The interviews lasted between 30 to 90 minutes. Transcripts were not sent to participants for checking. Following the

interviews, participants completed a questionnaire collecting demographic information, 10-item System Usability Scale (SUS) questions to evaluate usability, and acceptability questions adapted from the Acceptability Rating Scale for Decision-Aids (Ref 8,9). We refined the website iteratively during usability testing.

Measures

We used SUS, a widely used measure of subjective usability, in which each item is rated on a 5-point Likert scale (from "Strongly Disagree" to "Strongly Agree"). We calculated the total SUS score by summing the item scores and normalizing them to a 0–100 scale, where higher scores indicate better usability. A score above 68 indicates good usability (Ref 8). We hypothesized that *My Voice* will achieve a mean SUS score above the established threshold of 68, indicating good usability.

We tailored the acceptability survey for each participant group. Patients rated 6 items, caregiver rated 4 items and HCPs rated 8 items on a 4-point Likert scale ("Poor," "Fair," "Good," "Excellent"). All participants also rated the amount of information (too much, too little, just right), if they find My Voice useful (yes, no, unsure) and whether they will recommend My Voice to others (yes, no, unsure). Questions regarding acceptability were tailored for the specific stakeholder group (details in Supplementary table 3).

Data analysis

We transcribed the interviews and conducted thematic content analysis to identify key sub-themes and themes.(S3) The coding was managed on NVivo 11. Open coding was conducted, and themes were derived from subset of codes generated through the analysis. Although we achieved data saturation after coding 18 interviews, all transcripts were analysed. To ensure rigor and transparency, two team members independently coded the data. For Mandarin interviews, we translated the transcripts into English before analysis. We also summarized key suggestions to guide improvements to *My Voice*. We used descriptive statistics to summarize participants' demographic characteristics, SUS scores and proportion rating *My Voice* as acceptable.

Supplementary References

S1. Barnum CM. Usability testing essentials: Ready, set... test!: Morgan Kaufmann; 2020.
S2. Fan M, Lin J, Chung C, Truong KN. Concurrent Think-Aloud Verbalizations and Usability Problems. ACM Trans Comput-Hum Interact. 2019;26(5):Article 28.
S3. Elo S, Kyngäs H. The qualitative content analysis process. Journal of Advanced Nursing. 2008;62(1):107-15.

Supplementary Tables

Supplementary Table 1: Participant characteristics

Item	Patients (n = 9)	Caregivers (n = 5)	Healthcare providers (n = 10)
**Age, years mean (SD)**	69 (10.2)	61.8 (9)	40.3 (6.8)

**Gender, n (%)**			
Male	6 (66.7)	2 (40)	3 (30)
Female	3 (33.3)	3 (60)	7 (70)
**Ethnicity, n (%)**			
Chinese	3 (33)	2 (40)	
Non-Chinese	6 (67)	3 (60)	
**Marital Status, n (%)**			
Married	4 (44)	5 (100)	
Single (divorced/widowed/never married)	5 (56)	0 (0)	
**Education, n (%)**			
Primary or lower	4 (44)	2 (40)	
Secondary or higher	7 (66)	3 (60)	
**Duration of kidney failure, n (%)**			
< 5 years	1 (11.1)	0 (0)	
5 to < 10 years	4 (44.4)	3 (60)	
10 years and above	3 (33.3)	2 (40)	
Don't know	1 (11.1)	0 (0)	
**Relationship with patient n (%)**			
Spouse		4 (80)	
Others (relative)		1 (20)	
**Profession, n (%)**			
Nephrologist			3 (30)
Nurse			4 (40)
Social worker			2 (20)
Renal counsellor			1 (10)
**Experience with treating kidney failure patients, n (%)**			
< 5 years			2 (40)
5 to < 10 years			2 (40)
10 years and above			6 (60)
**Advance care planning (ACP) training**			
Yes			6 (60)
No			4 (40)

Supplementary Table 2: System Usability Scale score overall and by participant group

**System Usability Scale score**	**Patients (n = 9)**	**Caregivers (n = 5)**	**Healthcare providers (n = 10)**	**Overall (n = 24)**
Range (0 – 100)	52.5 - 90	45 - 85	57.5 – 97.5	45 – 97.5
Mean (SD)	71.9 (16.5)	69.5 (16)	83.5 (11.9)	76.2 (15.3)

Supplementary Table 3: Acceptability ratings of *My Voice*

Items	Patients n=9	HCPs n=10	Caregivers n=5
**Acceptability of Steps in the respective modules**	Good/excellent rating n (%)	Good/excellent rating n (%)	Good/excellent rating n (%)
About kidney failure and dialysis (Patient & caregiver step 1)	8 (89)	10 (100)	4 (80)
What happens when dialysis is stopped (Patient and caregiver step 2)	8 (89)	10 (100)	4 (80)
Understand what is important to you when it comes to your health (Patient step 3)	9 (100)	10 (100)	
Choosing a healthcare spokesperson (Patient step 4)	9 (100)	9 (90)	
Speak to your healthcare providers (Patient step	9 (100)	10 (100)	
Revisit *My Voice* (Patient step 6)	9 (100)	9 (90)	
Talk to your loved one (Caregiver step 3)		9 (90)	3 (60)
Support your loved ones (Caregiver step 4)		9 (90)	4 (80)
**Other items**	**n (%)**	**n (%)**	**n (%)**
Amount of information on website was			
Too little	0	0	2 (40)
Just right	9 (100)	10 (100)	3 (60)
My Voice is useful should you need to make decisions about dialysis in the future			
Yes	8 (89)	9 (90)	5 (100)
Unsure	1 (11)	1 (10)	0
Recommend *My Voice* website to others			
Yes	8 (89)	5 (100)	10 (100)
No	1 (11)	0	0
My Voice includes enough information to help them (i.e. patients) understand what is important to them			
Yes	9 (100)	10 (100)	
Value clarification question 'dialysis currently helps me to' is			
Easy to answer	7 (78)		
Difficult to answer	1 (11)		
Neither easy nor difficult to answer	1 (11)		

Items	Patients n=9	HCPs n=10	Caregivers n=5
Value clarification question ‘in the future, you may consider stopping dialysis if’ was			
Easy to answer	6 (67)		
Difficult to answer	2 (22)		
Neither easy nor difficult	1 (11)		
My Voice website is comparable to doing traditional advance care planning			
Yes		8 (80)	
No		2 (20)	
My Voice website is useful in helping you (caregivers) understand the role of the healthcare spokesperson			
Yes			5 (100)

Supplementary Table 4: Changes made to ‘My Voice’ as suggested by participants

Usability features	Suggestions	Implemented Yes/No	Comments
**Major changes**	Make *My Voice* a health care provider (HCP) guided tool	Yes	**HCP module was** created for HCPs to guide patients through the VCE. This module is similar to the patient module except that it allows HCPs to capture the open-ended discussion with patients in a free text box, which is also captured in the ‘My Voice’ document
	Include the option for palliative dialysis	Yes	**We revised Step 2** and **rescripted** video 2 to include topic of reducing frequency of dialysis per week (palliative dialysis) as a step before dialysis withdrawal. We also correspondingly added a quiz question on this topic. We changed step 2 title to ‘what are the options when dialysis becomes difficult to tolerate’.
	Make *My Voice* available in all local languages	Yes	***My Voice* was already available in English and Mandarin; we added Malay language website** option created with the content translated

			by a professional translation company.
**Visual & navigation**	Color code the correct answers in the quiz	No	To focus on empathetic communication, we did not highlight the right or wrong answers.
	Have a narration/voice option for text	No	Instead, increased font size and reduced wordiness to enable better reading
	Edit icon (to change and values) in *My Voice* summary page is confusing	Yes	Icon redesigned
	Increase font size of *My Voice* summary document	Yes	Font size increased
**Aesthetics**	Color code the navigation bar to show completed steps	Yes	Completed steps were color-coded green
	Separate first line in the quiz – good guess or good understanding from the text	Yes	Spaced out
	Improve the visual appeal of *My Voice* summary document	Yes	The template redesigned
**Phrasings in value clarifying exercise (VCE) section**	VCE answer options are many in some of the multiple-choice questions	No	Covered common values patients list out
	'Anything else' choice option for questions to be worded better	Yes	Added, please specify to make it clear
	'Be symptom free' as an option (for value clarifying questions) is not true	Yes	Changed to 'reduced symptoms'
	Unclear phrasing -stopping dialysis (*after the dialysis session, or totally stop)*	Yes	Term edited to 'stopping dialysis treatment'
**Others**	Indicate *My Voice* summary document is not a legal document	Yes	Added disclaimer to *My Voice* document- '*This document is not legally binding'*
